# Association of plasma iron with the risk of incident cancer in Chinese adults with hypertension: a nested case-control study

**DOI:** 10.3389/fonc.2023.1223579

**Published:** 2023-10-04

**Authors:** Hehao Zhu, Yaping Wei, Qiangqiang He, Yun Song, Lishun Liu, Yong Sun, Hao Zhang, Huiyuan Guo, Xiping Xu, Binyan Wang

**Affiliations:** ^1^ School of Science, China Pharmaceutical University, Nanjing, China; ^2^ College of Public Health, Shanghai University of Medicine and Health Sciences, Shanghai, China; ^3^ Shenzhen International Graduate School, Tsinghua University, Shenzhen, China; ^4^ Clinical Research Center, Shenzhen Evergreen Medical Institute, Shenzhen, China; ^5^ Department of Neurosurgery, People’s Hospital of Lianyungang City/The First Affiliated Hospital of Kangda College of Nanjing Medical University, Lianyungang, China; ^6^ College of Food Sciences and Nutritional Engineering, China Agricultural University, Beijing, China; ^7^ Key Laboratory of Precision Nutrition and Food Quality, Department of Nutrition and Health, China Agricultural University, Beijing, China

**Keywords:** plasma iron, cancer, hypertension, nested case-control study, digestive cancer

## Abstract

**Background:**

Iron is an essential element for organismal health but excessive iron is potentially toxic. However, few observational studies link plasma iron (PI) concentrations and cancer risk, and the results are inconsistent.

**Objective:**

This study aimed to explore the associations of PI concentrations with cancer risk in Chinese adults with hypertension.

**Methods:**

We conducted a nested, case-control study, including 223 pairs of incident cancer cases and matched controls from the China Stroke Primary Prevention Trial. The median time between blood sample collection and subsequent cancer event occurrence was 2.13 years. The odds ratio (OR) and 95% confidence interval (CI) for the risk of cancer by PI were estimated from multivariable conditional logistic regression models.

**Results:**

There was a nonlinear association between PI concentrations and total cancer risk. When compared with participants in tertile 2 of PI, the ORs of total cancer were 2.17 (95%CI: 1.25-3.85) and 1.29 (95%CI: 0.77-2.19) in participants in PI tertiles 3 and 1, respectively. Furthermore, higher PI was associated with increased digestive system cancer risk (OR=3.25, 95%CI:1.29-8.90), while lower PI was associated with increased risk of non-digestive system cancer (OR=3.32, 95%CI: 1.39-8.71). In a sensitivity analysis, the increases in total cancer risk or digestive system cancer risk were still observed with higher PI after excluding cancer cases occurring within the first year.

**Conclusion:**

Our results showed an increased risk of cancer related to higher PI or lower PI in Chinese adults with hypertension. Higher iron levels were linked to an increased risk of digestive system cancers, whereas lower iron levels were linked to an increased risk of non-digestive system cancers.

## Introduction

Cancer, which is increasing around the world, is expected to rank as the leading cause of mortality and the main barrier to life expectancy (1). Iron plays a vital role in cell replication, metabolism, and growth and is becoming more relevant in the research of chronic disease and cancer (2). However, iron status can be a double-edged sword, for excess iron status has been determined to be harmful to human health in that it generates a lot of free radicals, such as reactive oxygen species, in reaction with hydrogen peroxide (3). Excessive free radicals are a well-known cause of cell and tissue organ damage, and these processes may affect the development of cancer (4). One study showed that patients with hereditary hemochromatosis, a genetic iron disorder characterized by elevated plasma iron (PI) levels, had an increased risk of liver cancer (5).

A large number of studies have shown that abnormal iron homeostasis is one of the markers of cancer. PI has traditionally been used as a short-term marker of body iron status (6). The results on the relationship between blood iron and cancer risk have not always been consistent and no statistically significant associations (7) or positive associations (8, 9), as well as inverse associations (10), have been indicated. A cohort of 309,443 adults in Taiwan showed that high PI (≥120 mg/dL) is a marker of increased risk for total cancers, especially liver cancer (8). In contrast, a meta-analysis in a Chinese population found that PI levels were lower in patients with cervical cancer than in controls (10). A clinical trial also found that a reduction of iron stores was associated with a modestly decreased cancer risk and mortality (7). However, a large prospective cohort from Sweden reported no association between PI and cancer risk except for postmenopausal breast cancer (11). Additionally, one prospective study reported that the relationship between PI and nonskin cancers was converse between the sexes with a negative association in males and a positive association in females (12). Taken together, the evidence that links iron status with cancer has yielded mixed results.

Different associations between nutrients and cancers may occur in individuals with or without hypertension. All the previous studies were conducted in general populations. However, to date, there is a lack of data on cancer incidence associated with PI among patients with hypertension. Given the conflicting findings from epidemiologic studies, we hypothesize that the association between iron status and cancer risk is site-dependent and varies by population characteristics. In the current study, we prospectively examined the association between PI and total cancer and its subgroups in Chinese adults with hypertension and evaluated possible effect modifiers on the iron-cancer association.

## Materials and methods

### Study design and participants

The present study employed a nested, case-control design based on data from the China Stroke Primary Prevention Trial (CSPPT, clinicaltrials.gov identifier: NCT00794885). A detailed description of the cohort has been reported elsewhere (13). This multi-community, randomized, double-blind clinical trial aimed to evaluate the effectiveness of daily treatment with enalapril folic acid compared to enalapril alone in preventing first strokes and other outcomes, including cancer. The trial enrolled 20,702 adults with hypertension, aged 45 to 75 years, across thirty-two communities in China’s Jiangsu and Anhui provinces. It began on 19 May 2008 and concluded on 24 August 2013.

Participant follow-up involved phone or door-to-door visits, as well as clinic appointments, conducted every three months over a median period of 4.5 years. Throughout the follow-up period, the occurrence of endpoint events was documented. Hypertension was defined as self-reported use of antihypertensive medication, history of hypertension, or seated, resting systolic blood pressure of 140 mm Hg or higher, or diastolic blood pressure of 90 mm Hg or higher. The participants in the CSPPT had no history of physician-diagnosed stroke, myocardial infarction, heart failure, coronary revascularization, congenital heart disease, or cancer at the time of recruitment.

### Outcome assessment

Cancer was a pre-specified endpoint of the CSPPT and was the main outcome of our study. Cancer was diagnosed based on pathological findings. Original or photocopied pathological reports and original or photocopied medical records from hospitals were taken as evidence for pathological findings. In case pathological data was not available, two oncologists reviewed cases independently. Only if both of the physicians made the same clinical diagnosis based on clinical manifestations and examinations, cancer was diagnosed. An Endpoint Adjudication Committee, whose members were unaware of the treatment assignments, reviewed and adjudicated all cancer events independently.

### Selection of cases and controls

To mitigate confounding factors, a nested, case-control design was adopted. As shown in **Supplemental Figure 1**, among the 20,702 CSPPT participants, 232 patients were identified as having new, physician-diagnosed cancer until the end of the follow-up. Those participants who were alive and never developed cancer or cardiovascular disease during the follow-up period were matched with incident cancer cases in a 1:1 ratio. Matching criteria were age ( ± 1 year), sex, residence and treatment group. After excluding participants with missing iron data (n=4) and iron concentrations above 500 ug/dL(n=5), 223 cancer case-control pairs were obtained. The median time between blood sample collection and subsequent cancer event occurrence was 2.13 years. Among the 223 cancer patients, 123 cases were digestive cancers (including esophageal cancer, stomach cancer, liver cancer, pancreatic cancer, and colorectal cancer) and 100 cases were non-digestive cancers (including lung cancer and other non-digestive cancers).

### Data collection and assessment

At baseline, all study participants completed a standard questionnaire interview, including information on age, sex, education, occupation, smoking status, alcohol consumption, medical history, current medical condition, and medication intake. Height, weight, and seated blood pressure were measured by trained research staff according to standard protocol. Blood samples were collected at baseline. Serum homocysteine, fasting lipids, and glucose levels were measured using automatic clinical analyzers (Beckman Coulter) at the core laboratory of the National Clinical Research Center for Kidney Disease, Nanfang Hospital, Guangzhou, China. Serum vitamin B12 and folate levels were measured in a commercial laboratory using a chemiluminescent immunoassay (New Industrial), as published previously (13). PI, retinol, and 25-hydroxyvitamin D (25(OH)D) were measured by liquid chromatography with tandem quadrupole mass spectrometers (LC-MS/MS) in a commercial lab (Beijing DIAN Medical Laboratory) from August 2016 to July 2017 following standard lab protocol and vigorous quality control procedures.

### Statistical analysis

Continuous variables were expressed as mean ± standard deviation and compared using t-tests or median (75th percentile-25th percentile) and compared using rank-sum tests. Categorical variables were presented as number (percentage) and were compared using chi-square tests. To evaluate the association between PI and total cancer, subgroups (digestive cancers and non-digestive cancers), and four subtypes of cancers with sample sizes greater than 20 (esophageal cancer, gastric cancer, breast cancer, lung cancer), we utilized multivariate conditional logistic regression models. Here, PI was treated both as continuous variables, scaled to the standard deviation (SD), and as categorical variables (tertiles). Additionally, linear trend tests were conducted. To assess potential nonlinear associations, we employed restricted cubic spline regressions with 3 knots positioned at the 10th, 50th, and 90th percentiles. The reference point for these analyses was set at the median PI level observed in the population. If a nonlinear association had been identified, a segmented regression model was conducted to determine the breakpoint in this relationship. Subsequently, we proceed to further analyze the associations both below and above the identified breakpoints. The selection of adjustment variables was based on stepwise conditional logistic regression analysis and these were chosen based on those previously reported in the literature and included ten continuous variables: body mass index (BMI), baseline systolic blood pressure (SBP), fasting blood glucose, total cholesterol, triglycerides, high-density lipoprotein cholesterol (HDL-C), total homocysteine, vitamin B12, plasma retinol, and 25(OH)D; and two categorical variables: smoking status (ever vs. never) and drinking status (ever vs. never). In order to conduct a sensitivity analysis, we excluded cases diagnosed within one year after blood sampling. Furthermore, a multivariate conditional logistic regression model was employed. Interactions were examined by including the interaction terms in the logistic regression models. All analyses were performed in R software version 4.2.1 and two-tailed *P* < 0.05 was considered statistically significant.

## Results

### Characteristics of the participants

The analysis included 223 case-control pairs with PI measurements. Cancer cases included 123 cases of digestive system cancers (52 esophageal, 42 gastric, 18 colorectal, 7 liver, and 4 pancreatic cancers) and 100 cases of non-digestive system cancers (26 breast,26 lung, 9 lymphoma, 8 gynecologic, 7 bladder, and 24 cancers from other sites). The distribution of major cancer subtypes is presented in **Supplemental Table 1**. **Table 1** shows the baseline characteristics of the total cases and controls. Median values of PI were 140.1 μg/dl in patients with cancer and 134.1 μg/dl in controls. There were no significant differences between total cancer cases and controls for any of the variables.

**Table 1:** Baseline characteristics by case-control status.

Characteristics	Total cancers		
	Cases (n=223)	Controls (n=223)	*P*
Age, y	61.8 (56.9,67.5)	61.8 (57.0,67.5)	0.987
Male, No (%)	117 (52.5)	117 (52.5)	1.000
Body mass index, kg/m^2^	23.8 (21.6,26.4)	24.2 (21.7,26.4)	0.485
No smoking, No. (%)	121 (54.3)	133 (59.6)	0.251
No alcohol drinking, No. (%)	138 (61.9)	139 (62.3)	0.922
Blood pressure, mmHg			
Baseline SBP	161.3 (150.7,175.3)	160.7 (152.7,174.7)	0.502
Baseline DBP	91.3 (84.0,100.0)	92.0 (87.3,100.0)	0.244
Treatment group, No. (%)			
Enalapril	114 (51.1)	114 (51.1)	1.000
Enalapril-folic acid	109 (48.9)	109 (48.9)	
Center			
Anqing	84 (37.7)	84 (37.7)	1.000
Lianyungang	139 (62.3)	139 (62.3)	
Laboratory results			
Serum total cholesterol, mmol/L	5.3 (4.6,6.1)	5.3 (4.6,6.0)	0.670
Serum triglycerides, mmol/L	1.4 (1.0,2.0)	1.4 (1.1,1.9)	0.971
Serum HDL-C, mmol/L	1.3 (1.1,1.6)	1.3 (1.1,1.5)	0.235
Fasting glucose, mmol/L	5.2 (4.8,5.8)	5.4 (4.8,6.1)	0.089
Serum creatinine, mmol/L	66.7 (56.2,76.9)	66.5 (55.7,80.8)	0.727
Serum total homocysteine, mmol/L	12.8 (10.7,15.9)	13.0 (10.7,16.5)	0.649
Serum folate, ng/mL	8.5 (5.6,11.0)	8.4 (5.9,11.6)	0.655
Serum vitamin B12, pg/mL	381.6 (322.7,475.6)	391.7 (318.7,491.4)	0.681
Plasma 25(OH)D, ng/mL	20.7 (16.7,25.2)	21.0 (16.4,25.9)	0.555
Plasma retinol, ug/dL	64.9 (51.2,85.1)	67.6 (54.4,82.4)	0.503
Plasma iron, ug/dL	140.1 (106.6,178.5)	134.1 (105.9,166.1)	0.268

Continuous variables are presented as mean ± standard deviation or median (interquartile range) a. Categorical variables are presented as number (percentage). DBP: diastolic blood pressure; SBP: systolic blood pressure; HDL-C: high-density lipoprotein cholesterol; 25(OH)D: 25-hydroxyvitamin.

### Association of baseline PI and the risk of total cancer and its subtypes

**Figure 1:**
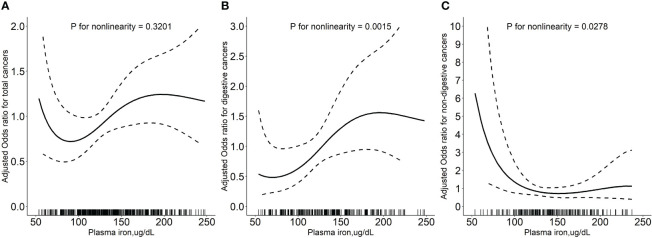
Relationship of plasma iron with the risk of total cancers **(A)**, digestive system cancers **(B)**, and non-digestive system cancers **(C)**. Adjusted for baseline systolic blood pressure, body mass index, smoking status, drinking status, fasting blood glucose, serum total cholesterol, triglycerides, high-density lipoprotein cholesterol, total homocysteine, vitamin B12, plasma retinol, and 25-hydroxyvitamin D CI: confidence interval; OR: odds ratio.

Overall, there was a nonlinear association between PI and total cancer with an increased risk of total cancer in participants who had higher or lower levels of PI (**Figure 1A**). Higher levels of PI were associated with an increased risk of digestive system cancers (**Figure 1B**), while lower levels of PI were associated with an increased risk of non-digestive system cancers (**Figure 1C**). Breakpoint estimates were provided by segmented regression models with digestive system cancers (breakpoint=165.6, 95%CI: 42.2-289.0) and non-digestive system cancers (breakpoint=145.6, 95%CI: 115.1-176.2). Below the breakpoint for non-digestive system cancers, there was a negative association between PI and cancer risk (OR=0.97, 95%CI: 0.96-0.99, *P*=0.007, **Supplemental Table 2**). After adjusting for potential confounders, compared to participants in PI tertile 2 (114.1 to <150.4 ug/dL), those in tertile 3 (≥150.4 ug/dL) conferred a significantly increased risk for total cancer (OR: 2.17, 95%CI: 1.25-3.85, *P*=0.007) while those in tertile 1(<114.1 ug/dL) also showed an increased risk, but it was not statistically significant (OR: 1.29, 95%CI: 0.77-2.19, *P*=0.340). A substantially higher risk of digestive system cancer was seen for those with higher levels of PI (≥150.4 ug/dL vs 114.1 to <150.4 ug/dL, OR=3.25, 95%CI: 1.29-8.90, *P*=0.016). However, a substantially higher risk of non-digestive system cancer was seen for those with lower levels of PI (<114.1 ug/dL vs 114.1 to <150.4 ug/dL, OR=3.32; 95%CI: 1.39-8.71; *P*=0.010, **Table 2**). Similar results were seen after excluding cases who had been diagnosed within 1 year after the blood draw (**Supplemental Table 3**). Further investigation into the relationship between PI and the risk of specific cancer sites revealed that patients with higher PI levels had a significantly increased risk of gastric cancer (≥150.4 ug/dL vs 114.1 to <150.4 ug/dL, OR=22.68, 95%CI: 2.28-225.83, *P*=0.008, **Supplemental Table 4**).

**Table 2:** Association between baseline iron levels and the risk of total cancer and subgroups.

Plasma iron, ug/dL	N	Cases (%)	Crude model		Adjusted model	
			OR (95%CI)	*P*	OR (95%CI)	*P*
Total cancer						
Per SD increase			1.11 (0.91,1.36)	0.300	1.10 (0.89,1.37)	0.363
Tertiles						
T1 (<114.1)	142	68 (47.9)	1.20 (0.73,1.99)	0.465	1.29 (0.77,2.19)	0.340
T2 (114.1 to <150.4)	134	59 (44.0)	1.00 (1.00,1.00)	Ref.	1.00 (1.00,1.00)	Ref.
T3 (≥150.4)	170	96 (56.5)	1.92 (1.14,3.28)	0.015	2.17 (1.25,3.85)	0.007
* P* for trend				0.083		0.091
Digestive cancers						
Per SD increase			1.31 (1.00,1.76)	0.061	1.42 (1.02,2.05)	0.048
Tertiles						
T1 (<114.1)	87	37 (42.5)	0.92 (0.47,1.82)	0.807	0.98 (0.43,2.24)	0.965
T2 (114.1 to <150.4)	70	32 (45.7)	1.00 (1.00,1.00)	Ref.	1.00 (1.00,1.00)	Ref.
T3 (≥150.4)	89	54 (60.7)	2.59 (1.19,6.01)	0.020	3.25 (1.29,8.90)	0.016
* P* for trend				0.009		0.021
Non-digestive cancers						
Per SD increase			0.94 (0.70,1.25)	0.659	0.73 (0.48,1.08)	0.122
Tertiles						
T1 (<114.1)	55	31 (56.4)	1.84 (0.87,4.05)	0.118	3.32 (1.39,8.71)	0.010
T2 (114.1 to <150.4)	64	27 (42.2)	1.00 (1.00,1.00)	Ref.	1.00 (1.00,1.00)	Ref.
T3 (≥150.4)	81	42 (51.9)	1.54 (0.76,3.23)	0.239	1.52 (0.63,3.71)	0.353
* P* for trend				0.711		0.101

Odds ratios of cancer in relation to plasma iron were calculated using conditional logistic regression models. Each subgroup analysis adjusted for systolic blood pressure, body mass index, smoking, alcohol drinking, fasting blood glucose, serum total cholesterol, triglycerides, high-density lipoprotein cholesterol, total homocysteine, vitamin B12, plasma retinol, and 25-hydroxyvitamin D; CI, confidence interval; OR, odds ratio; T1, tertile 1; T2, tertile 2; T3, tertile 3.

### Stratified analyses on the associations between plasma iron and cancer risk

To explore potential effect modifiers for the association between PI and cancer risk, 11 subgroup analyses were conducted stratified by age, sex, BMI, treatment group, smoking status, drinking status, total cholesterol, fasting glucose, total homocysteine, plasma retinol, and 25(OH)D (**Table 3**). Higher PI levels were significantly associated with an increased risk of total cancer in those with plasma retinol ≥67.0 ug/dL (median) and the *P*-value for the interaction and the false discovery rate (FDR) adjusted *P*-value were 0.047 and 0.404, respectively. None of the other variables significantly modified the association between plasma PI and total cancer.

**Table 3:** Stratified analyses by potential effect modifiers for the association between plasma iron and risk of incident cancer.

Subgroups	T1	T2	T3	OR (95%CI) (T1 vs T2)	OR (95%CI) T3 vs T2	*P* for interaction	*P^a^*
Age, years						0.975	0.975
<65	42/46	38/48	62/47	1.12 (0.60,2.09)	1.77 (0.95,3.27)		
≥65	26/28	21/27	34/27	1.31 (0.57,2.99)	1.89 (0.80,4.49)		
Sex						0.928	0.975
Male	28/32	26/36	63/49	1.08 (0.51,2.29)	2.02 (1.00,4.08)		
Female	40/42	33/39	33/25	1.38 (0.69,2.77)	1.85 (0.86,3.96)		
Body mass index, kg/m^2^						0.824	0.975
<25	46/44	30/35	40/28	1.21 (0.62,2.36)	1.69 (0.80,3.57)		
≥25	22/30	29/40	56/46	1.05 (0.49,2.25)	1.97 (0.98,3.95)		
Treatment group						0.858	0.975
Enalapril	35/39	29/39	50/36	1.16 (0.58,2.34)	2.19 (1.07,4.48)		
Enalapril-folic acid	33/35	30/36	46/38	1.14 (0.56,2.32)	1.36 (0.67,2.80)		
Smoking status						0.372	0.682
Never	48/47	38/52	35/34	1.46 (0.79,2.71)	1.42 (0.72,2.80)		
Ever	20/27	21/23	61/40	0.81 (0.34,1.95)	2.23 (1.02,4.90)		
Drinking status						0.332	0.682
Never	53/50	42/55	43/34	1.49 (0.81,2.74)	1.66 (0.86,3.18)		
Ever	15/24	17/20	53/40	0.87 (0.33,2.34)	2.49 (1.01,6.16)		
Total cholesterol, mmol/L						0.113	0.404
<5.3 (median)	36/50	29/36	47/29	0.91 (0.46,1.83)	1.95 (0.94,4.03)		
≥5.3	32/24	30/39	49/45	1.76 (0.82,3.80)	1.95 (0.94,4.04)		
Fasting glucose, mmol/L						0.862	0.975
<5.6	51/52	42/49	59/36	1.18 (0.65,2.12)	1.99 (1.05,3.76)		
≥5.6 or diabetes	17/22	17/26	37/38	1.14 (0.44,2.98)	1.60 (0.70,3.68)		
Total homocysteine, mmol/L						0.135	0.404
<15.0	53/45	46/55	59/48	1.39 (0.77,2.51)	1.35 (0.74,2.45)		
≥15.0	15/29	13/20	37/26	0.65 (0.23,1.85)	2.70 (1.02,7.14)		
Plasma retinol, ug/dL						0.047	0.404
<67.0 (median)	44/33	36/40	35/34	1.58 (0.81,3.09)	1.17 (0.58,2.36)		
≥67.0	24/41	23/34	61/40	0.87 (0.41,1.86)	2.64 (1.29,5.44)		
25 (OH)D, ng/mL						0.147	0.404
<20.9 (median)	28/39	34/35	52/35	0.73 (0.36,1.50)	1.77 (0.88,3.55)		
≥20.9	40/35	25/40	44/39	1.82 (0.90,3.67)	1.71 (0.84,3.50)		

P^a^ represents FDR adjusted P for interaction. The odds ratios of cancer in relation to plasma iron were calculated using unconditional logistic regression models. Each subgroup analysis adjusted for age, sex, treatment group, study site, systolic blood pressure, body mass index, smoking status, drinking status, systolic blood pressure, fasting blood glucose, serum total cholesterol, triglycerides, high-density lipoprotein cholesterol, total homocysteine, vitamin B12, plasma retinol, and 25-hydroxyvitamin D except itself; 25(OH)D: 25-hydroxyvitamin D; CI: confidence interval; OR: odds ratio; T1: tertile 1; T2: tertile 2; T3: tertile 3; FDR: false discovery rate.

## Discussion

This nested case-control study suggests a nonlinear association between PI and the risk of total cancer, with higher cancer risk associated with both higher and lower iron levels in Chinese adults with hypertension. Moreover, we found that higher iron levels (≥150.4 ug/dL) were linked to an increased risk of digestive system cancers, whereas lower iron levels (<114.1 ug/dL) were linked to an increased risk of non-digestive system cancers. Our findings demonstrate that the association of iron with cancer might differ according to tumor location.

The mean PI level in our study was 144.2 ug/dL, which was remarkably higher than that observed in the United States (83.88 ug/dL), with data from the National Health and Nutrition Examination Survey (NHANES) from 2007 to 2016 (14). To avoid reverse causation bias and potential confounding bias, we additionally excluded cancer cases that had occurred within the first year, as well as adjusted for potential confounders, and found that the positive association was still observed. Our results support the idea that body iron plays a key role in human carcinogenesis (2). Some studies have reported sex differences in the association of PI with cancer risk (12, 15). A prospective cohort study of US adults suggested that participants with PI above 140 µg/dl had a significantly increased risk of cancer mortality (15) and this association was stronger in females. However, no sex difference was observed in our study.

The relationship between PI concentrations and digestive system cancer risk observed in this study is in line with the prevailing hypothesis that higher body iron is associated with an increased risk of liver cancer (16). A systematic review showed that PI was positively correlated with liver cancer risk (HR: 2.47; 95%CI:1.31-4.63) (16). In a large cohort study in Taiwan, it was revealed that PI levels higher than 120 µg/dL or lower than 60 µg/dL were associated with a 25% increase and 18% increase, respectively, in all cancer incidence with 60 to 79 ug/dL as the reference level (8), primarily with liver cancer. A Mendelian randomization study in a European cohort suggested that a genetically high iron status was positively associated with liver cancer (17). For other digestive tract tumors, Milde et al. observed that adenocarcinoma colorectal patients had significantly higher levels of PI as compared to the age-matched control group (18). Results from the European Prospective Investigation into Cancer and Nutrition (EPIC) based on 481,419 individuals and 137 incident cases of esophageal adenocarcinoma, showed a statistically significant positive association of esophageal adenocarcinoma risk with heme iron and processed meat intake (19). Recent research suggests that red meat (high iron content) intake could be a risk factor for gastric cancer (20). However, one study from a large European population found that high body iron stores as measured by PI and ferritin decreased the risk of gastric cancer (21). Another study also found that high iron stores may increase the risk of colorectal cancer, whereas low iron stores may be an early sign of occult stomach cancer (22).

Data on the association between PI and non-digestive system cancers are limited and inconclusive. One study reported a dual role for iron in breast cancer where a proangiogenic environment mediated by iron deficiency and pro-oxidant conditions associated with iron accumulation could lead to a high incidence of breast cancer (23). A recent meta-analysis found that high levels of PI were associated with an increased risk of breast cancer (24). Nonetheless, some studies have reported results that were similar to ours (10, 25). A meta-analysis in a Chinese population found that PI levels were lower in patients with cervical cancer than in controls (10). A previous publication also reported that iron deficiency is known to be associated with bladder cancer (25). A study by Yuan et al. suggested that genetically high iron status was inversely associated with brain cancer based on 48,972 individuals of European-descent (17). However, a meta-analysis showed no association between PI levels and lung cancer risk (26). Epidemiological data on the association of iron status with other non-digestive system cancers are limited and scarce. More studies are needed to better elucidate this possible relation. Our study has provided some new insights. We demonstrated that the relationship between PI levels and cancer risk varies for different cancer subtypes, with a positive association for digestive system cancers and negative association for non-digestive system cancers.

Plausible reasons for the positive correlation between PI and digestive system cancers could be explained by the fact that the gastrointestinal tract is the major site of nutrient digestion and absorption, and it is prone to oxidative damage (27). Accumulated iron has been implicated in the risk of cancer through iron-catalyzed free radical-mediated oxidative stress and subsequent DNA damage, and iron also functions as a nutrient that fosters the growth and development of cancer cells. Additionally, it has been postulated that iron insufficiency could lead to oxidative deoxyribonucleic acid damage, thereby increasing the risk of cancer (28, 29). Elemental iron is crucial for the proper functioning of enzymes involved in cell respiration, energy metabolism, DNA synthesis and repair, signaling, and many more. Iron is an important trace element required for the formation of hemoglobin and myoglobin and is a key component in immune cell proliferation. Therefore, iron is both essential and potentially toxic. However, the specific mechanism of iron metabolism in different types of tumor cells is not yet fully understood and the interaction mechanism of retinol and iron should be explored further.

There are several strengths of this study. The data are directly derived from a large cohort study, with strict quality control measures. Furthermore, our study utilized a matched design to minimize potential data bias as much as possible. First, we focused our analysis on PI, one of the biomarkers of iron status, and the analysis did not include other measures that are thought to more closely reflect levels of iron in the body, such as ferritin levels. As PI is a relatively inexpensive and widely available test, tests for high PI status could be routinely conducted and interpreted in daily practice, making our results clinically relevant. Moreover, Chua et al. suggest that circulating iron may be more related to breast carcinogenesis than stored iron (ferritin) (12). A second study limitation was that the average follow-up time from the time of PI testing was 2.13 years, which may be too short a time for assessing cancer development. A third limitation is that the conclusions from our study may not apply to other populations as the participants were patients with hypertension. The differences in findings might be attributed to residual confounding by other confounders, such as unhealthy dietary patterns or other lifestyle behaviors.

In conclusion, our study provides evidence of a nonlinear relationship between iron status and overall cancer. Considering the small number of cancer cases, additional studies with larger sample sizes are warranted to verify our findings. Furthermore, the effects of iron on different cancer types need to be explored. The results of this study have important clinical implications with regard to the excessive supplement of iron and iron deficiency.

## Data availability statement

The datasets presented in this article are not readily available because Data described in the article, code book, and analytic code will be made available upon request pending approval. Requests to access the datasets should be directed to Zhu Hehao, zhuhehao@foxmail.com.

## Ethics statement

The studies involving humans were approved by The CSPPT was approved by the Ethics Committee of the Institute of Biomedicine, Anhui Medical University, Hefei, China (FW A assurance number: FW A00001263). The studies were conducted in accordance with the local legislation and institutional requirements. The participants provided their written informed consent to participate in this study.

## Author contributions

The authors’ responsibilities were as follows— HeZ and YW: data curation, writing original draft; QH: supervision; YuS and LL: audited the data; XX: conception and conducted research; HG, YoS and HaZ: helped with copyediting; BW: had primary responsibility for the final content of the manuscript. All authors read and approved the final manuscript.
