# Supplementary material for: Association of plasma iron with the risk of incident cancer in Chinese adults with hypertension: a nested case-control study
Source: Front Oncol. 2023 Oct 4;13:1223579. doi: 10.3389/fonc.2023.1223579 (PMC10583576; doi:10.3389/fonc.2023.1223579)
Supplement: Supplementary file 1 [file DataSheet_1.docx]

Association of plasma iron with the risk of incident cancer in Chinese adults with hypertension: a nested case-control study

Hehao Zhu^1,^*, Yaping Wei^2,3,^*, Qiangqiang He^4,5^, Yun Song^5^, Lishun Liu^4,5^, Ziyi Zhou^4,5^, Jianping Li^6^, Yan Zhang^6^, Yong Sun^7^, Hao Zhang^2,3^, Huiyuan Guo^2,3^, Xiping Xu^2,3^, and Binyan Wang^5 ✉^

*** Corresponding Authors**:

**Binyan Wang, M.D.**

Shenzhen Evergreen Medical Institute, Shenzhen, 518051, China

Email: [binyanwang163@163.com](mailto:binyanwang163@163.com)

**
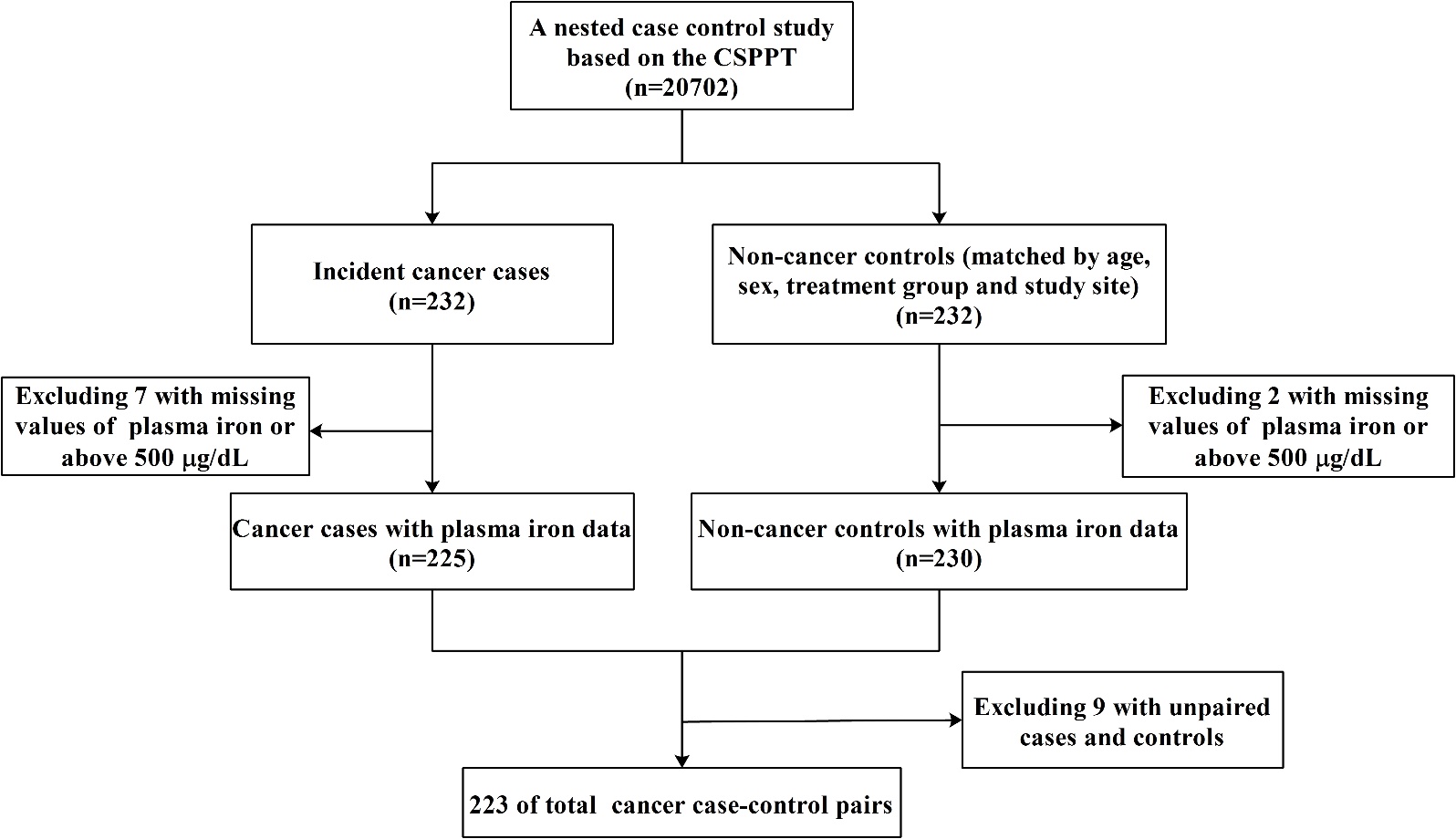
**

**Supplemental Figure 1. Flow chart of the study participants using a nested case-control design.**

**Supplemental Table 1. Distribution of major cancer subtypes.**

| **Cancer types** | **N** | **%** |
| --- | --- | --- |
| **Overall** |  |  |
| **Digestive cancers** | 123 |  |
| Esophageal cancer | 52 | 23.32 |
| Gastric cancer | 42 | 18.83 |
| Colorectal cancer | 18 | 8.07 |
| Liver cancer | 7 | 3.14 |
| Pancreatic cancer | 4 | 1.79 |
| **Non-Digestive cancers** | 100 |  |
| Breast cancer | 26 | 11.66 |
| Lung cancer | 26 | 11.66 |
| Lymphoma cancer | 9 | 4.04 |
| Gynecologic cancer | 8 | 3.59 |
| Bladder cancer | 7 | 3.14 |
| Other cancer | 24 | 10.76 |

**Supplemental Table 2. Associations between plasma iron and cancer risk below and above the breakpoint.**

| **Plasma iron, ug/dL** | **N** | **Cases (%)** | **Crude model** | | **Adjusted model** | |
| --- | --- | --- | --- | --- | --- | --- |
|  |  |  | **OR (95%CI)** | ***P*** | **OR (95%CI)** | ***P*** |
| **Digestive cancers** |  |  |  |  |  |  |
| Below the breakpoint (165.6) |  |  |  |  |  |  |
| Per SD increase |  |  | 1.01(1.00,1.02) | 0.171 | 1.01(1.00,1.02) | 0.071 |
| Category |  |  |  |  |  |  |
| T1(<102.3) | 60 | 27(45.0) | 1.00(1.00,1.00) | Ref. | 1.00(1.00,1.00) | Ref. |
| T2(102.3 to <132.5) | 60 | 26(43.3) | 0.93(0.45,1.92) | 0.854 | 0.88(0.39,1.97) | 0.753 |
| T3(≥132.5) | 61 | 33(54.1) | 1.44(0.70,2.95) | 0.318 | 1.78(0.75,4.24) | 0.190 |
| *P* for trend |  |  |  | 0.315 |  | 0.216 |
| Above the breakpoint (165.6) |  |  |  |  |  |  |
| Per SD increase |  |  | 1.00(0.99,1.01) | 0.890 | 1.00(0.99,1.01) | 0.774 |
| Category |  |  |  |  |  |  |
| T1(<188.1) | 22 | 13(59.1) | 1.00(1.00,1.00) | Ref. | 1.00(1.00,1.00) | Ref. |
| T2(188.1 to <219.9) | 21 | 15(71.4) | 1.73(0.48,6.18) | 0.398 | 10.00(1.11,90.34) | 0.040 |
| T3(≥219.9) | 22 | 9(40.9) | 0.48(0.14,1.59) | 0.230 | 1.28(0.24,6.91) | 0.772 |
| *P* for trend |  |  |  | 0.226 |  | 0.943 |
| **Non-digestive cancers** | |  |  |  |  |  |
| Below the breakpoint (145.6) |  |  |  |  |  |  |
| Per SD increase |  |  | 0.98(0.97,1.00) | 0.028 | 0.97(0.96,0.99) | 0.007 |
| Category |  |  |  |  |  |  |
| T1(<106.3) | 38 | 20(52.6) | 1.00(1.00,1.00) | Ref. | 1.00(1.00,1.00) | Ref. |
| T2(106.3 to <125.9) | 37 | 19(51.4) | 0.95(0.38,2.35) | 0.912 | 0.72(0.26,2.02) | 0.535 |
| T3(≥125.9) | 38 | 16(42.1) | 0.65(0.26,1.62) | 0.359 | 0.45(0.16,1.28) | 0.134 |
| *P* for trend |  |  |  | 0.36 |  | 0.134 |
| Above the breakpoint (145.6) |  |  |  |  |  |  |
| Per SD increase |  |  | 1.00(0.99,1.01) | 0.931 | 1.00(0.99,1.01) | 0.747 |
| Category |  |  |  |  |  |  |
| T1(<166.0) | 29 | 15(51.7) | 1.00(1.00,1.00) | Ref. | 1.00(1.00,1.00) | Ref. |
| T2(166.0 to <194.1) | 29 | 14(48.3) | 0.87(0.31,2.44) | 0.793 | 1.13(0.33,3.91) | 0.847 |
| T3(≥194.1) | 29 | 16(55.2) | 1.15(0.41,3.23) | 0.792 | 0.80(0.22,2.90) | 0.740 |
| *P* for trend |  |  |  | 0.793 |  | 0.737 |

ORs of cancer in relation to plasma iron were calculated using unconditional logistic regression models. Each subgroup analysis adjusted for systolic blood pressure, body mass index, smoking, alcohol drinking, fasting blood glucose, serum total cholesterol, triglycerides, high-density lipoprotein cholesterol, total homocysteine, vitamin B12, plasma retinol, and 25-hydroxyvitamin D; T1: tertile 1; T2: tertile 2; T3: tertile 3.

**Supplemental Table 3. Association between baseline plasma iron levels and the risk of total cancer and subgroups excluding cancer cases diagnosed in the first year.**

| **Plasma iron, ug/dL** | **N** | **Cases (%)** | **Crude model** | | **Adjusted model** | |
| --- | --- | --- | --- | --- | --- | --- |
|  |  |  | **OR (95%CI)** | ***P*** | **OR (95%CI)** | ***P*** |
| **Total cancer** |  |  |  |  |  |  |
| Per SD increase |  |  | 1.19(0.96,1.49) | 0.129 | 1.22(0.96,1.56) | 0.109 |
| Category |  |  |  |  |  |  |
| T1(<114.1) | 124 | 50(40.3) | 0.83(0.48,1.41) | 0.491 | 0.90(0.51,1.57) | 0.709 |
| T2(114.1 to <150.4) | 125 | 50(40.0) | 1.00(1.00,1.00) | Ref. | 1.00(1.00,1.00) | Ref. |
| T3(≥150.4) | 148 | 74(50.0) | 1.92(1.11,3.42) | 0.022 | 2.13(1.17,3.99) | 0.016 |
| *P* for trend |  |  |  | 0.041 |  | 0.037 |
| **Digestive cancers** |  |  |  |  |  |  |
| Per SD increase |  |  | 1.53(1.07,2.28) | 0.026 | 1.56 (1.01,2.58) | 0.056 |
| Category |  |  |  |  |  |  |
| T1(<114.1) | 70 | 28(40.0) | 0.75(0.35,1.58) | 0.444 | 0.83(0.32,2.08) | 0.684 |
| T2(114.1 to <150.4) | 57 | 27(47.4) | 1.00(1.00,1.00) | Ref. | 1.00(1.00,1.00) | Ref. |
| T3(≥150.4) | 63 | 40(63.5) | 3.25(1.27,9.50) | 0.020 | 3.57 (1.17,12.38) | 0.032 |
| *P* for trend |  |  |  | 0.003 |  | 0.010 |
| **Non-digestive cancers** | |  |  |  |  |  |
| Per SD increase |  |  | 1.07(0.77,1.50) | 0.678 | 1.00(0.66,1.53) | 0.987 |
| Category |  |  |  |  |  |  |
| T1(<114.1) | 42 | 22(52.4) | 1.54(0.66,3.73) | 0.322 | 2.84(1.00,9.08) | 0.060 |
| T2(114.1 to <150.4) | 54 | 23(42.6) | 1.00(1.00,1.00) | Ref. | 1.00(1.00,1.00) | Ref. |
| T3(≥150.4) | 62 | 34(54.8) | 1.77(0.80,4.12) | 0.166 | 2.29(0.87,6.54) | 0.103 |
| *P* for trend |  |  |  | 0.776 |  | 0.122 |

ORs of cancer in relation to plasma iron were calculated using unconditional logistic regression models. Each subgroup analysis adjusted for systolic blood pressure, body mass index, smoking, alcohol drinking, fasting blood glucose, serum total cholesterol, triglycerides, high-density lipoprotein cholesterol, total homocysteine, vitamin B12, plasma retinol, and 25-hydroxyvitamin D; T1: tertile 1; T2: tertile 2; T3: tertile 3.

**Supplemental Table 4. Association between baseline plasma iron levels and the risk of** **different cancers.**

| **Plasma iron, ug/dL** | **N** | **Cases (%)** | **Crude model** | | **Adjusted model** | |
| --- | --- | --- | --- | --- | --- | --- |
|  |  |  | **OR (95%CI)** | ***P*** | **OR (95%CI)** | ***P*** |
| **Esophageal cancer** |  |  |  |  |  |  |
| Per SD increase |  |  | 1.00(1.00,1.01) | 0.284 | 1.00(0.99,1.01) | 0.410 |
| Category |  |  |  |  |  |  |
| T1(<114.1) | 26 | 11(42.3) | 0.94(0.29,3.10) | 0.919 | 0.55(0.12,2.39) | 0.422 |
| T2(114.1 to <150.4) | 30 | 14(46.7) | 1.00(1.00,1.00) | Ref. | 1.00(1.00,1.00) | Ref. |
| T3(≥150.4) | 48 | 27(56.3) | 1.93(0.57,6.51) | 0.291 | 1.77(0.42,7.42) | 0.432 |
| *P* for trend |  |  |  | 0.245 |  | 0.127 |
| **Gastric cancer** | |  |  |  |  |  |
| Per SD increase |  |  | 1.01(1.00,1.02) | 0.110 | 1.01(1.00,1.02) | 0.047 |
| Category |  |  |  |  |  |  |
| T1(<114.1) | 40 | 17(42.5) | 1.22(0.42,3.57) | 0.718 | 1.42(0.39,5.14) | 0.592 |
| T2(114.1 to <150.4) | 19 | 7(36.8) | 1.00(1.00,1.00) | Ref. | 1.00(1.00,1.00) | Ref. |
| T3(≥150.4) | 25 | 18(72.0) | 7.44(1.40,39.43) | 0.018 | 22.68(2.28,225.83) | 0.008 |
| *P* for trend |  |  |  | 0.020 |  | 0.011 |
| **Breast cancer** |  |  |  |  |  |  |
| Per SD increase |  |  | 1.00(0.99,1.01) | 0.563 | 0.99(0.98,1.01) | 0.329 |
| Category |  |  |  |  |  |  |
| T1(<114.1) | 18 | 10(55.6) | 1.20(0.33,4.34) | 0.780 | 1.00(0.25,4.08) | 0.998 |
| T2(114.1 to <150.4) | 18 | 9(50.0) | 1.00(1.00,1.00) | Ref. | 1.00(1.00,1.00) | Ref. |
| T3(≥150.4) | 16 | 7(43.8) | 0.83(0.23,3.01) | 0.780 | 0.49(0.10,2.33) | 0.367 |
| *P* for trend |  |  |  | 0.548 |  | 0.483 |
| **Lung cancer** |  |  |  |  |  |  |
| Per SD increase |  |  | 1.00(0.99,1.02) | 0.745 | 0.99(0.97,1.02) | 0.527 |
| Category |  |  |  |  |  |  |
| T1(<114.1) | 14 | 6(42.9) | 0.53(0.09,3.21) | 0.487 | 1.43(0.13,15.78) | 0.771 |
| T2(114.1 to <150.4) | 13 | 7(53.9) | 1.00(1.00,1.00) | Ref. | 1.00(1.00,1.00) | Ref. |
| T3(≥150.4) | 25 | 13(52.0) | 1.17(0.17,8.05) | 0.871 | 1.68(0.09,32.20) | 0.730 |
| *P* for trend |  |  |  | 0.447 |  | 0.978 |

ORs of cancer in relation to plasma iron were calculated using unconditional logistic regression models. Each subgroup analysis adjusted for systolic blood pressure, body mass index, smoking, alcohol drinking, fasting blood glucose, serum total cholesterol; T1: tertile 1; T2: tertile 2; T3: tertile 3.
